# Supplementary material for: Reproductive number of coronavirus: A systematic review and meta-analysis based on global level evidence
Source: PLoS One. 2020 Nov 11;15(11):e0242128. doi: 10.1371/journal.pone.0242128 (PMC7657547; doi:10.1371/journal.pone.0242128)
Supplement: Attachment — Submitted filename: Response to reviewers.docx [file pone.0242128.s002.docx]

Authors response to reviewers’ comments

| **Comments and suggested changes** | **Changes made and authors’ reply** |
| --- | --- |
| **Editor** |  |
| 1. Given that the authors are approaching a meta-analysis on such a specific topic, they should have made it clear that COVID-19 is not the virus (which is, indeed, called SARS-CoV-2). COVID-19 stands for Coronavirus disease-19, and is the disease caused by the virus, so the basic reproduction number cannot be referred to the disease, but to the etiological agent. Please do correct everywhere in the title, abstract, manuscript, tables and figures, where appropriate. No additional revision process can be carried on in the absence of this basic requirement. | We have made these changes throughout the manuscript. |
| 2. The data search dates back to April 10, 2020. Given the rapidly evolving epidemiological scenario, the search needs to be updated to eventually include any new published study. | We have searched data again on September 15, 2020, and updated the analyses accordingly. |
| 3.  "Data extraction and Analysis" paragraph, page 8, lines 4-5: "For the studies where more than one reproductive number reported based on different assumptions, we calculated the average reproductive number. This calculated average reproductive number was then used to give summary estimate". This approach is not entirely correct. When multiple estimates are available for the same study, a pooled estimate should be computed from the available data using a fixed-effect meta-analysis. Then the resulting estimate should be included in the overall meta-analysis (see as an example Flacco et al Heart 2020; heartjnl-2020-317336). | We have edited relevant sentences accordingly to fix this issue. We change the term ‘*average reproductive number*’ into ‘*pooled estimate of the reproductive number*’. Besides, ‘*Data extraction and Analysis’* section has been divided into ‘*Data extraction’* and ‘*Statistical analysis*’ separately. The revised sentence read-  *Meta-analysis was used for the studies that consistently reported the reproductive number and its 95% confidence interval. We first use the fixed-effect meta-analysis to get a pool reproductive number for the studies which reported more than one reproductive number for a country calculated based on different assumptions. Later this pooled estimate was used to give a summary estimate of the reproductive number. We used the random-effect meta-analysis to estimate the summary reproductive number.* |
| 4. Table 2: some results from a meta-regression are listed, but they are not commented in the results, nor reported in the methodology: please do clarify. | We have added the following sentences to refer to Table 2 results-  *Later we explored the sources of heterogeneity through sub-groups analysis across the selected studies’ characteristics. These include the country for which the reported reproductive number was estimated, the method and model that were used to estimate the reproductive number, total number of case that was considered to estimate the reproductive number and type of reproductive number that was reported. We also assessed the publication bias through visual inspection of the funnel plot and Egger’s regression asymmetry test. The trim-and-fill procedure was used when evidence of publication bias was found.*  We have added following sentences in the results section for explaining the Table 2 result.  *However, we found, through using the meta-regression, these differences were only significant across the countries of the reported reproductive number and the methods used to estimate the reproductive number.* |
| 5. Page 7, last line: "Meta-analysis [WAS] then used to give an average estimate of the reproductive number". The estimate resulting from a meta-analytical approach is not simply an average and should not be defined as such. It is a pooled, or summary estimate. Please do correct everywhere in the text where appropriate. | We have fixed this issue throughout the manuscript. |
| 6.  At least two previous meta-analyses were published on the same topic (He W et al. J Med Virol. 2020 May 29:10.1002/jmv.26041. doi: 10.1002/jmv.26041; Alimohamadi Y et al. J Prev Med Public Health. 2020 May;53(3):151-157. doi: 10.3961/jpmph.20.076). Please discuss your results also in light of these previous findings. | We have provides a comprehensive discussion regarding the advantage of this then previous published studies in the Introduction as well in the Discussion section. In the Introduction section the relevant sentences read-  *To settle these disagreements on the reported reproductive number and know the current situation of infection, a summary estimate of the reproductive number is important. However, of the three studies that have been provided summary reproductive number so far were limited in several areas and did little to settle these disagreements [12–14]. For instance, they reported a summary estimate of the basic reproductive number without considering the net reproductive number and the time-dependent reproductive number. However, it is around 10 months that have already been gone since the first infection of the coronavirus in December 2019 and all countries have been imposed several prevention measures. Therefore, the estimation of the basic reproductive number was available only in a few studies of which these summary estimates were based. Moreover, these studies were also failed to address the heterogeneity of their estimated reproductive number though it was found higher [12–14].* |
| 7. Minor issues:  - The length of the introduction could be easily reduced, as many of the reported information on the hystory of the pandemic and on the main characteristic of the SARS-CoV-2 virus are already well known, and could be reduced to one or two short paragraphs. Please go straight to the point.  - The manuscript needs a careful language editing, as there are some errors and misspellings (see as an example: "Meta-analysis WAS then used to give an average estimate of the reproductive number.", or "the estimated reproductive number was around double (..) in outside of China than China"). | We have edited the introduction section and reduced its length- now it is straighter than it was in the previous version.  We have also checked the language carefully and fixed the errors. |
| **Reviewer #1** |  |
| Abstract: Aim and study methodology is fairly stated but need to touch on some special feature on the topic to provoke read full paper. | We have revised abstract- please have look at the revised version. |
| Introduction: Introduction could have been more effective. Context of the study is moderately stated but lacks enough logical progression. Importance of this study should benefit the readers and provide a rough solution which will be convinced in discussion section. Please refer to the previous meta-analyzes(for example: Estimate of the Basic Reproduction Number for COVID-19: A Systematic Review and Meta-analysis. Alimohamadi, Yousef et al. J Prev Med Public Health 2020; 53(3): 151-157. DOI: <https://doi.org/10.3961/jpmph.20.076>) and mention the relative advantages of your work over previous studies. | We have revised the introduction section. Please look have a look at the manuscript. We also provided the advantages of this study over the previously published studies. |
| Figure 1 needs to be self explanatory with stand alone graphics. a and b are not shown in the fig. | To respond to the editor’s comment, we have deleted this figure. |
| Methodology: Selection, presentation and sampling of various studies were fairly explained. As you mentioned in the introduction section there are at least 3 types of R, but according to the search strategy, it is not clear which type of R has been studied. Has subgroup analysis been performed for different types of R? If yes, why aren't the results presented? If possible, some insights from the authors about possible reasons for those larger outliers (around 6), or any particular characteristics of those studies (e.g. small sample, earlier stage, or particular region?) | We have edited this section to clarify these issues. We considered each type of reproductive number for this study. We have made their estimate available in this version, please see Table 2. We have also explained their results in the result section which read-  *Variations were also found across the type of reported reproductive numbers- the time-dependent reproductive number was found around double (R,4.42; 95% CI, 3.05-6.40) than the net reproductive number (R,1.95; 95% CI, 1.63-2.34). However, we found, through using the meta-regression, these differences were only significant across the countries of the reported reproductive number and the methods used to estimate the reproductive number.*  We also provided provide a broad description of the source of variations of the estimated reproductive number. Please have a look at the discussion section. |
| Results: Figures and tables needs to be self explanatory with stand alone graphics. Please provide the results of the quality assessment of each study in the table 1. Please provide the detailed results of the funnel plot , Egger’s test and Trim-and Fill procedure. | We have edited the tables and figures times to made themself-explanatory.  We have now added the summary quality assessment results in Table 1. However, we keep their details results in the supplementary table as it was.  We have added funnel plots in the manuscript. We also have added details results for each test. Please see the related figure. |
